# Proactive psychiatry of addiction: toward the normalization of early prevention

**DOI:** 10.1192/j.eurpsy.2023.152

**Published:** 2023-07-19

**Authors:** V. Hendriks

**Affiliations:** LUMC Curium Dept. Child and Adolescent Psychiatry, Parnassia Group; Leiden University Medical Centre (LUMC), Leiden University, The Hague, Netherlands

## Abstract

**Abstract:**

Most substance use disorders (SUDs) emerge in adolescence and young adulthood. Early interventions in young people may reduce the risk and severity of SUD and other mental disorders. However, we are not able to reach our young people early enough for prevention and treatment, and when we do, they often already show a high concentration of comorbid mental disorders and signs of a chronic intermittent course of SUD. Hence, we must reach our addicted young patients at an earlier stage, when symptoms are still mild or transient, or perhaps even before that – when they only show precursors of possible dysfunction.

One of the most prominent precursors of dysfunction is our ability – or lack thereof – to control or "self-regulate" our behaviors, cognitions, and emotions. Many scientists argue that poor self-regulation is perhaps the core determinant of the development of mental health disorders, including addiction. Several prospective general population studies have shown that poor childhood self-control early in life is a strong predictor of many negative outcomes later in life, up to 20 to 30 years later in adulthood. Although correlational in nature, these findings suggest that early childhood interventions that are deliberately aimed at improving self-regulation may be effective in preventing these negative life outcomes, and that early prevention and intervention targeted at improving self-control may reduce the risk of a broad array of psychiatric and social problems, including addiction. Indeed, several recent large-scale systematic reviews have suggested that self-regulation skills are malleable and can be learned through instruction and practice, and perhaps most so in the early years, roughly around 3 to 6 years, when there is a steep increase in learning curve, when the plasticity of the brain is still high, and when self-regulation skills are still very much in development.

This presentation provides an overview of the rationale and study findings of early prevention of substance use disorders and other mental health disorders. In terms of broad prevention, much can be gained by widespread, consistent implementation and normalization of early prevention at the pre- and elementary school level.

**Disclosure of Interest:**

None Declared

